# Serum and follicular fluid thyroid hormone levels and assisted reproductive technology outcomes

**DOI:** 10.1186/s12958-019-0529-0

**Published:** 2019-11-07

**Authors:** Yun Ying Cai, Na Lin, Lan Ping Zhong, Hui Juan Duan, Yun Hua Dong, Ze Wu, Heng Su

**Affiliations:** 1 Medical School, Kunming University of Science and Technology, Kunming, 650500 Yunnan Province China; 2 Department of Endocrinology, The Affiliated Hospital of Kunming University of Science and Technology, Kunming, 650500 Yunnan Province China; 3 Reproductive Medicine Center, The Affiliated Hospital of Kunming University of Science and Technology, Kunming, 650500 Yunnan Province People’s Republic of China

**Keywords:** Thyroid hormones, Follicular fluid, Controlled ovarian hyperstimulation, Thyroid autoimmunity

## Abstract

**Objective:**

The objective ofthis study was to assess the association between thyroid hormone (TH) levels in follicular fluid (FF) and serum and to determine whether THs impact assisted reproductive technology (ART) outcomes.

**Methods:**

This study enrolled 299 women undergoing ART. Blood samples were drawn on the day of human chorionic gonadotrophin (HCG) administrationand analysed for thyroid-stimulating hormone (TSH), thyroxine(T4), triiodothyronine(T3),free T4 (fT4),free T3(fT3), thyroid peroxidase antibody (TPOAb) and thyroglobulin antibody (TGAb) levels. FF was obtained on the oocyte pick up (OPU) day and analysed forTSH, T4, T3, fT4, fT3, TPOAb, TgAb and estradiol levels.

**Results:**

(1) There were significant positive correlations between serum and FF TH and thyroid autoantibody levels. Statistically significant differences were discovered in serum and FF levels of TSH (*p* ≤ 0.001), T4 (*p* ≤ 0.001), T3 (*p* ≤ 0.001), TPOAbs (*p* ≤ 0.001) and TGAbs (*p* = 0.021).

(2) Serum T4 levels [121.9(104.8,140.8) vs 114.1(98.6,130.6) nmol/l, *p* = 0.026], serum fT4 levels[(19.0(17.7,21.8) vs 18.6(17.0,20.1) pmol/l, *p* = 0.026], serum T4/T3 ratios [62.5 (55.7, 66.2) vs 59.4 (53.4, 64.9), *p* = 0.029], FF fT4 levels [19.0(17.5,21.3) vs 18.1(16.8,19.9) pmol/l, *p* = 0.009] and FF T4/T3 ratios [52.6 (46.4, 57.3) vs 50.0 (43.7, 53.1), *p* = 0.004] were significantly higher in the successful pregnancy group than the implantation failure group.

(3) Spearman’s rank correlation analysis revealed positive associations of both the FF T4/T3 ratio and serum TSH levels with the numbers of retrieved oocytes (total or MII) and embryos (fertilized, cleavage, and good quality).

**Conclusions:**

TH levels in FF are strongly correlated with those in serum on the HCG day, and THs on the HCG day may affect ART outcomes.

## Introduction

Thyroid hormones (THs) are related to infertility and multiple adverse neonatal and maternal consequences [1, 2]. Recently, many studies have evaluated the relationship between ART outcomes and thyroid function [3–5]. A growing body of literature debates what constitutes “normal” gestational and preconceptional thyroid function and treatment cut-offs [4, 5]. The latest meta-analysis on preconception subclinical hypothyroidism (SCH), which included 14,846 participants, found no significant differences in ART-related outcomes between different thyroid-stimulating hormone (TSH) level groups when the TSH cut-off was set to 2.5 mIU/L. However, when a broader TSH cut-off range (3.5–5 mIU/L) was used, the miscarriage rate was higher in the preconception SCH group than in the normal group [4]. On the other hand, several studies of euthyroid infertile women undergoing intrauterine insemination (IUI) [6–8], found no differences in TSH levels among different IUI outcome groups. In addition, most previous studies categorized women with no history of thyroid disease and normal TSH levels as euthyroid; TH levels and thyroid antibody status were not known.

Follicular fluid (FF) supports the acquisition of development competence in oocytes [9, 10], and provides the important microenvironment for oocyte maturation. Changes in the FF levels of hormonesand metabolites have been reported to influence oocyte quality, early embryo development, and subsequent pregnancy [11, 12]. Since biological effects of THs are regulated by deiodinase (DIO) in peripheral tissue [13, 14], serum TH levels do not always predict tissue-specific effects in target organs, and local THs may play a direct role in physiological functions. Although the presence of THs in human FF was verified in 1993 [15], the concentration of THs in FF has been analysed in only a small number of studies [16, 17]. One preliminary observational study found that fT4 in FF were higher in infertile patients than in the healthy population [18]. However, data from literature are not sufficiently clear to definitely state the relationship between serum and FF TH levels and the outcomes of assisted reproductive technology (ART),such as in vitro fertilisation (IVF).

Previous studies used only serum TSH as a biomarker to evaluate thyroid function. The aim of our study was to compare the relationship of serum and FFTHs withcycle parameters and ART outcomes…

## Materials and methods

This study was approved by the Ethics Committee of the First People’s Hospital of Yunnan Province and was carried out according to good clinical practices. Informed consent was obtained from each patient.

### Patients and sample collection

This prospective study involved a cohort of subfertile women who underwent one IVF or IVF-intracytoplasmic sperm injection (ICSI) cycle. For this study, two hundred and ninety-nine subfertile women were enrolled. A total of 165 FFsamples were collected for the final analysis. TSH levels were determined at the first visitto our clinical center. Only patients with normal TSH and a baseline Day 3 FSH level <10 IU/L were included in the study. We excluded women with pre-exiting thyroid disease or medication usage(*N* = 30) and those withcancelled cycles (*N* = 104)). Participants were followed to determine early pregnancy outcomes (3 months). This analysis exclusively refers to the first treatment cycle.

### IVF procedure and sample collection

For controlled ovarian stimulation (COS), we used one of two protocols, treatment with the long gonadotropin-releasing hormone agonist triptorelin (Decapeptyl 1.25 mg, Ferring Co, Kiel, Germany) for 2 weeks, starting on cycle day 21 or the antagonist protocol (Cetrotide 0.25 mg/ml, cetrorelix acetate, Merck Serono, Frankfurt, Germany) in combination with recombinant FSH (Gonal F Serono, Aubonne, Switzerland). Human chorionic gonadotrophin(HCG) (250 μg of Gonal f,EMD Serono, Aubonne, Switzerland) was administered when more than three follicles reached a diameter of > 18 mm. Oocyte retrieval was performed by the transvaginal ultrasound-guided approach, 36 h after HCG injection. Blood samples were drawn in the morning on the day of HCG administration. FF was collected only from the first puncture of oneovary. Samples were processed and stored at − 20 °C until analysis. Based on availabe literature and data, we selected the total number of blastomeres andMII oocytes as potential determinants of oocyte maturation. Clinical pregnancy was defined as ultrasonographic demonstration of a vital embryo within an intrauterine gestational sack 4–5 weeks after embryo transfer. The implantation rate was calculated as the ratio of the number of gestational sacks identified at this time to the number of embryos transferred.

### Laboratory analysis

The levels of TSH, triiodothyronine (T3), T4, free T3(fT3), free T4(fT4), thyroid peroxidase antibody (TPOAb) and thyroglobulin antibody (TGAb) were measured with electro-chemiluminescence (ECL) immunoassays (CobasElesys 601, Roche). The assays had the following reference ranges and intra-assay coefficients of variation (CVs): TSH, 0.27–4.2 mIU/l and 1.57–4.82%; T3, 1.3–3.1 nmol/l and 1.71–5.97%; T4, 66–181 nmol/l and 2.36–6.12%; fT3, 3.1–6.8 pmol/l and 2.42–5.61%; fT4, 12–22 pmol/l and 2.24–6.34%; TPOAb ≤34 IU/ml and 1.98–6.7%; and TGAb, ≤115 IU/ml and 1.64–5.37%.

### Statistical analysis

Quantitative values are expressed as the mean ± SD or as the median and interquartile range, as appropriate. Student’s t-test was used to analyse continuous data with a normal distribution. Wilcoxon Rank-Sum test (nonparametric analysis) was used to evaluate continuous data without a normal distribution; chi-square analysis was used for categorical data with large cell counts, and Fisher’sexact test was used to evaluate categorical data with small cell counts,.A *p*-value ≤0.05 was considered statistically significant.

## Results

### Relationship and difference between serum and follicular fluid thyroid hormones

Our results showed significant positive correlation between serum THlevels and FF TH levels (TSH: *r* = 0.876, *p* ≤ 0.001;T4: *r* = 0.788, *p* ≤ 0.001; T3: *r* = 0.727, *p* ≤ 0.001;fT4:*r* = 0.853, *p* ≤ 0.001; fT3:*r* = 0.702, *p* ≤ 0.001). Significant differences in TSH (2.22 ± 1.13vs2.73 ± 1.43 mIU/l, *p* ≤ 0.001),T4(118.57 ± 22.6vs105.85 ± 21.5 nmol/l, *p* ≤ 0.001) and T3 levels (1.97 ± 0.33vs2.10 ± 0.32 nmol/l, *p* ≤ 0.001) were found between serum and FF. Moreover,fT4 and fT3 followed the same trend, but the differences did not reach statistical significance.

### Relationship and difference between serum and follicular fluid thyroid autoantibodies

Our results showed a significant correlation between serum and FF TPOAbs(*r* = 0.808, *p* ≤ 0.001) and TGAbs(*r* = 0.601, *p* ≤ 0.001).

TPOAb [15.27 (8.94, 19.9) vs 9.37 (5, 12.7) IU/ml, *p* ≤ 0.001] and TGAb levels [17.6 (14.8, 20.5) vs 15.6 (13.7, 20.3), *p* = 0.021] were higher in serum than in FF.

No statistically significant differences in serum TSH (2.53 ± 1.03 vs 2.22 ± 1.15 mIU/ml, *p* = 0.762), T4(116.2 ± 10.63 vs 118.81 ± 23.48 nmol/l, *p* = 0.941),T3(1.93 ± 0.12vs1.98 ± 0.34 nmol/l, *p* = 0.461), fT4 (19.55 ± 2.7 vs 19.16 ± 3.11 pmol/l, *p* = 0.169) and fT3 levels (4.68 ± 0.32 vs 4.55 ± 0.73 pmol/l, *p* = 0.154) were found between patients with and without thyroid autoantibodies.

### Serum and follicular fluid parameters andART characteristics and outcomes

We observedslightly higher serum T4 levels [121.9(104.8,140.8) vs 114.1(98.6,130.6) nmol/l, *p* = 0.026], serum fT4 levels[(19.0(17.7,21.8) vs 18.6(17.0,20.1) pmol/l, *p* = 0.026], serum T4/T3 ratios [62.5 (55.7, 66.2) vs 59.4 (53.4, 64.9), *p* = 0.029], FF fT4 levels [19.0(17.5,21.3) vs 18.1(16.8,19.9) pmol/l, *p* = 0.009] and FF T4/T3 ratios [52.6 (46.4, 57.3) vs 50.0 (43.7, 53.1), *p* = 0.004] in women with a successful pregnancy than in women with implantation failure in the respective treatment cycle. In terms ofTSH,T3, fT3, TPOAbs andTGAbs in serum and FF, there were no significant differences between the two groups (Table 1).

**Table 1:** Clinical and pretreatment parameters, follicular fluid and serum concentrations of hormones in 165 patients undergoing ovarian stimulation

Variables	Successful Pregnancy (*N* = 72)	Implantation Failure (*N* = 93)	*P* value
Clinical and treatment parameters			
Age (yrs)	31 (28,34)	34 (28,36)	NS
BMI (kg/m2)	22.5 (20.6,25.2)	20.8 (18.9,22.6)	≤0.001
Basal FSH	3.63 (1.95,6.01)	4.22 (1.79,7.11)	NS
AMH (ng/ml)	3.56 (1.78,3.91)	3.46 (2.25,4.13)	NS
Oocytes (total)	13.5 (8.8,16.8)	10 (7,14)	0.007
Oocyte (MII) Fertilization rate,%	10.5 (5.5,15) 65.9%	8 (6,10) 56.6%	0.002 ≤0.001
Etiology of infertility(%)			0.395
Female infertility	75%	77.4%	
Both male and female infertility	16.7%	9.7%	
Infertility of unknown cause	8.3%	12.9%	
Cleavage rate,%	98%	99.5%	NS
Serum (day HCG) hormone levels			
TSH (mIU/l)	1.86 (1.27,3.08)	1.95 (1.54,2.95)	NS
T4(nmol/l)	121.9 (104.8,140.8)	114.1 (98.6,130.6)	0.026
T3(nmol/l)	1.98 (1.74,2.22)	1.98 (1.75,2.13)	NS
fT4(pmol/l)	19.0 (17.7,21.8)	18.6 (17.0,20.1)	0.026
fT3(pmol/l)	4.75 (4.25,4.93)	4.5 (4.08,4.8)	NS
TPO Ab (IU/ml)	15.1 (9.17,23.22)	15.5 (8.94,17.32)	NS
TG Ab (IU/ml)	16.6 (13.86,22.2)	14.4 (13.3,19.9)	NS
T4/T3	62.5 (55.7,66.2)	59.4 (53.4,64.9)	0.029
Follicular fluid hormone levels			
TSH (mIU/l)	2.12 (1.41,3.57)	2.41 (2.03.3.23)	NS
T4(nmol/l)	112.3 (93.7124.5)	102.6 (88.6116.8)	NS
T3(nmol/l)	2.07 (1.88.2.22)	1.98 (1.75,2.13)	NS
fT4(pmol/l)	19.0 (17.5,21.3)	18.1 (16.8,19.9)	0.009
fT3(pmol/l)	4.67 (4.33,5.0)	4.19 (4.20,4.88)	NS
TPO Ab (IU/ml)	8.4 (5.0,13.9)	10.1 (5.0,12.7)	NS
TG Ab (IU/ml)	17.5 (13.98,24.38)	17.6 (14.9,20.3)	NS
T4/T3	52.6 (46.4,57.3)	50.0 (43.7,53.1)	0.004

Note: Values are Mean ± SD, median and interquartile range, or n (%) as indicated

*NS* nonsignificant,which means *P ≥ 0.05*

*FF* follicular fluid, *BMI* body mass index, *AMH* anti-Mullerian hormone

Spearman’s rank correlation analysis showed positive associations of both the FF T4/T3 ratio and serum TSH levels withthe number of retrieved oocytes (total or MII) and the number of embryos (fertilized, cleavage, and good quality). These associations were slightly stronger for serum TSH than for FF T4/T3 (Table 2). On the other hand, the positive correlations observed between TSH and the number of good quality oocytes were stronger for serum than for FF (serum TSH *r* = 0.41, *p* ≤ 0.001; FF TSH *r* = 0.31, *p* ≤ 0.001).

**Table 2:** Spearman rank correlation analysis of associations between FF and serum thyroid hormone levels and clinical and hormonal parameters

	TSH		T4		T4/T3		fT4	
	Serum	FF	Serum	FF	Serum	FF	Serum	FF
Oocytes (total)	0.0165 (*r* = 0.186)	0.478	0.676	0.0474 (*r* = 0.155)	0.313	0.043 (*r* = 0.159)	0.08	0.356
Oocyte (MII)	0.008 (*r* = 0.205)	0.103	0.476	0.005 (*r* = 0.216)	0.713	0.03 (*r* = 0.166)	0.191	0.874
Fertilized embryos	0.019 (*r* = 0.182)	0.251	0.195	0.003 (*r* = 0.228)	0.109	≤0.001 (*r* = 0.253)	0.478	.624
Cleavage embryos	≤0.001 (*r* = 0.271)	0.0726	0.173	0.004 (*r* = 0.226)	0.08	≤0.005 (*r* = 0.271)	0.42	0.68
Good quality embryos	≤0.001 (*r* = 0.41)	≤0.001 (*r* = 0.31)	0.633	0.2	0.02 (*r* = 0.18)	0.003 (*r* = 0.227)	0.596	0.908
E_2_ (HCG day)	≤0.001 (*r* = 0.324)	0.01 (*r* = 0.203)	0.003 (*r* = −0.28)	0.01 (*r* = −0.20)	0.004 (*r* = − 0.226)	0.08	≤0.001 (*r* = − 0.428)	≤0.001 (*r* = − 0.428)
E_2_ (FF)	0.552	0.575	0.789	0.302	0.916	0.89	0.273	0.302

Note:*FF* follicular fluid, *E2* oestradiol, *HCG* human chorionic gonadotropin

No statistically significant correlation wasfound between the number of retrieved oocytes or fertilized oocytes and the levels of thyroid autoantibodies in serum or FF (data not show).

Strong negative correlations of serum and FF fT4 levels with serum oestradiol (E2) were observed, but no such correlations were observed with FF E2 levels. The opposite correlation patterns were found between serum E2 levels andserum and FF TSH levels.

BMI had a statistically significant impact on ART outcome (*p* ≤ 0.001). Different aetiologies of infertility (male, female, both male and female, and unknown cause) did not have a statistically significant impact on ART outcome (*p* = 0.395).

## Discussion

The present study verified the presence of THs and thyroid autoantibodies in the FF of women undergoing ART and assessed the impact of these factors on embryonic development and ART outcomes. We demonstrated the presence of TSH, T4, T3, fT4, fT3 and thyroid autoantibodies in FF, and estimated their impact on fertilization and embryo development during ART. Our study highlighted that the majority of patients have FF TH levels within the normal serum range [15, 16] We also observed a significant positive correlation between serum and FF TH levels, indicating that the majority of THs detected in FF seem to be derived from peripheral blood and enter follicles through theca interna cells. Moreover, we detected significantly higher concentrations of T3 in FF than in serum, whereas T4 concentrations were higher in serum than in FF, which is in line with the findings of previous studies [15, 17]. Our work also showedthat the T4/T3 ratio was much lower in FF than in blood, which supportsthe presence of an ovarian 5′-monodeiodinase system in FF capable of generating T3 (ovary- generated T3) by outer ring deiodination of T4 [17]. We also detected thyroid autoantibodies in FF, but the levels were much lower than in serum. The results suggest that the blood-follicle barrier is a permselective barrier for thyroid autoantibodies.

The pivotal role of THs in several aspects of female reproduction have been well documented by several investigators. THs may impact folliculogenesis [19–21], ovarian steroidogenesis [22], and endometrial receptivity [23]. There is evidence that thyroid function is associated with pregnancy outcome, particularly in IVF. Despite treatment, women with hypothyroidism may have lower chances of pregnancy success after IVF [24]. SCH may also impact reproduction, and treatment of women with SCH has been shown to improve IVF outcomes [25, 26], including increasing the rates of implantation, clinical pregnancy, and delivery.

Because of the strong associations of SCH with subfertility, the definition of euthyroidism in subfertile women is currently a topic of debate. TSH is considered the most sensitive test for thyroid function, so currently, studies investigating the association between SCH and infertility are based on serum TSH levels. In the present study, we showed no difference in TSH levels in euthyroid women undergoing ART among different IVF outcome groups. Our results are in line with those recently reported by Karmon AE et al. [6–8]. In the group of euthyroid women undergoing infertility treatment with ART, the authors observed no significant differences in clinical pregnancy or delivery rates among euthyroid infertile women with different preconceptional TSH levels after IUI.

However, evidence is lacking to support the use of T4 or fT4 to categorize euthyroid infertile women undergoing IVF and to predict pregnancy outcomes. It is unknown whether distinctions can be made among TH values within the normal range of TSH and the chances of certain fertility outcomes. In the current study, the major differences between women achieving pregnancy and those who experienced implantation failure were in serum T4, fT4 levels and the T4/T3 ratio, This discrepancy among TSH,T4 and fT4 levels might be explained by the physiology: COS-induced hyperestrogenism directly reduces serum fT4 levels by stimulating thyroid binding globulin (TBG) production, whereas the increase in serum TSH levels is achieved by a negative feedback loop [27, 28]. Taken together, these data suggest that when there is a rapid increase in TBG leading to high variability in fT4 levels, the changes in TSH lags. Our study results show that the determination of fT4 levels and the T4/T3 ratio in serum on the day of OPU, together with TSH, maybe have greater prognostic value than TSH alone.

A growing body of literature indicates that the hormonal follicular milieu, which includes anti-Mullerian hormone and inhibin B, is correlated with reproductive outcome after IVF [29]. However, few studies have examined the possible association between FF THs and IVF outcomes. Our results are the first to show a link among the FF T4/T3 ratio, embryonic developmental competence and successful pregnancy. Embryo quality is an important predictor of ART success. As expected,the successful pregnancy group had a significantly higher mean number of blastomeres, MII oocytes and retrieved oocytes than the implantation failure group. Significant correlations were found between the numbers of retrieved oocytes and fertilized oocytes and serum TSH levels and the FF T4/T3 ratio. Kamron’s study advocates that thyroid function in subfertile women could indicate an insufficient capacity for basic reproductive functions such as oocyte quality, ovulation, fertilization and implantation [30]. Free TH supplementation (50 ng/ml of T3 and T4) led to significant increases in blastocyst formation and hatching rates in bovine embryos [31], and in the expansion rate of the blastocoel cavity of cryopreserved bovine embryos. Although an exact mechanism cannot be assumed, it is noteworthy that thyroid dysfunction may impact IVF outcomes, possibly at the level of oocyte quality.

No statistically significant difference was found between groups in the levels of thyroid autoantibodies in serum and FF. There was no statistically significant correlation between the number of retrieved oocytes or fertilized oocytes and the levels of thyroid autoantibodies in serum and FF. These findings support the theory that thyroid autoantibodies in FF do not affect oocyte number, oocyte maturation, orquality or preimplantation embryos during ART [32, 33]. However, other studies which only focus on serum TAI have found a lower fertilization rate in the thyroid autoimmunity (TAI)-positive group in the TAI-negative group(63 vs 72% [34] and 64.3 vs 74.6% [35]). One of the limitations of our study and other study included FF TAI to analysis is the relatively small number of TAI-positive patients. Our study included 165 (15 TAI positive) patients, while Medenica’s study included 52 (26 TAI positive) patients [32]. In addition, a previous study showed that thyroid antibodies cancross-react with zona pellucida and granulosa cells due to molecular mimicry [36].

Our results revealed significant negative correlations between serum E2 and fT4 in serum and FF. Not surprisingly, we also found a significant positive correlation between serum E2 and serum TSH. which could be explained by the COS-specific changes in oestrogen levels. A previous study showed that COS could lead to hyperestrogenism, through a rise in TBG, subsequently leading to a decrease in fT4 [27]. Our results support the theory that the adaptation of maternal THmetabolism to hyperestrogenism during pregnancy may be important for embryo survival [30, 31]. Similarly, COS-specific changesmight put stress on hypothalamus-pituitary-thyroid axis in a very short time and manifest as gestational thyroid disease, which subsequently creates a suboptimal environment during the early phase of implantation, which may increase the risk for IVF failure.

The use of a population of infertile women undergoing ART has some limitations. First, our outcomes were limited to cycle parameters and clinical pregnancy. Therefore, no conclusions can be drawn on any other obstetric or fetal end points. Second, whether these findings are generalizable to women without known fertility problems is still under debate. Additionally, a strong association between TH levels and pregnancy rate could not be found.

In conclusion, thyroid autoantibodies and mostTHs present in FF are not generated in the FF, but rather enter from the blood. Thyroid autoantibodies do not directly impact oocytes and embryos during ART, but on the OPU day,the T4/T3 ratios in both serum and FF were significantly higher in the group of women who became pregnant than in those who did not conceive. Our results highlight the importance of the adaptation of THmetabolism during COS.

Moreover, one point of practical importance regarding our study results is the confirmation of a strong correlation between serum and FF TH levels,which suggests that the complicated determinations of FF TH levels may not be necessary because serum TH levels provide the same information. Further studies with more samples should be performed to confirm our findings and to elucidate the relationships among ovarian stimulation protocols, oocyte quality, clinical pregnancy rate and longitudinal changes of THs levels during COS.

## Data Availability

The datasets used and/or analysed during the current study available fromthe corresponding author on reasonable request.
